# Seizure Susceptibility in *Drosophila melanogaster* is Reduced with CBD and *Z. jujube* treatment

**DOI:** 10.17912/micropub.biology.001799

**Published:** 2025-12-09

**Authors:** Zakary Kubitz, Jonathan Goedken, Brylee O'Neill, Luke DeWaard, Jack Kinzer, Josette Knutson, Avery Mize, Stephanie Toering Peters

**Affiliations:** 1 Wartburg College, Waverly, Iowa, United States

## Abstract

Seizures have a bidirectional relationship to sleep deprivation; sleep loss increases seizure susceptibility and duration, while seizures disrupt sleep (Mituzaite et al., 2021; Konduru et al., 2021). This study tested whether two treatments, cannabidiol (CBD) and *Zizyphus jujube* extract, reduced seizure activity in *Drosophila melanogaster* . We hypothesized the treatments would decrease seizure duration and increase seizure latency in sleep-deprived bang-sensitive ( *sesB ^9Ed-4^* ) and temperature-sensitive ( *sei ^P^* ) mutants, respectively. Sleep-deprived *sesB ^9Ed-4^* flies treated with *Z. jujube* showed reduced seizure duration and both treatments increased seizure latency in sleep-deprived *sei ^P^* flies. This demonstrates CBD and *Z. jujube* have anti-convulsant activity in the *Drosophila* model.

**Figure 1. Seizure susceptibility of sesB 9Ed-4 and sei P flies in response to sleep deprivation and treatment with CBD and Z. jujube:**
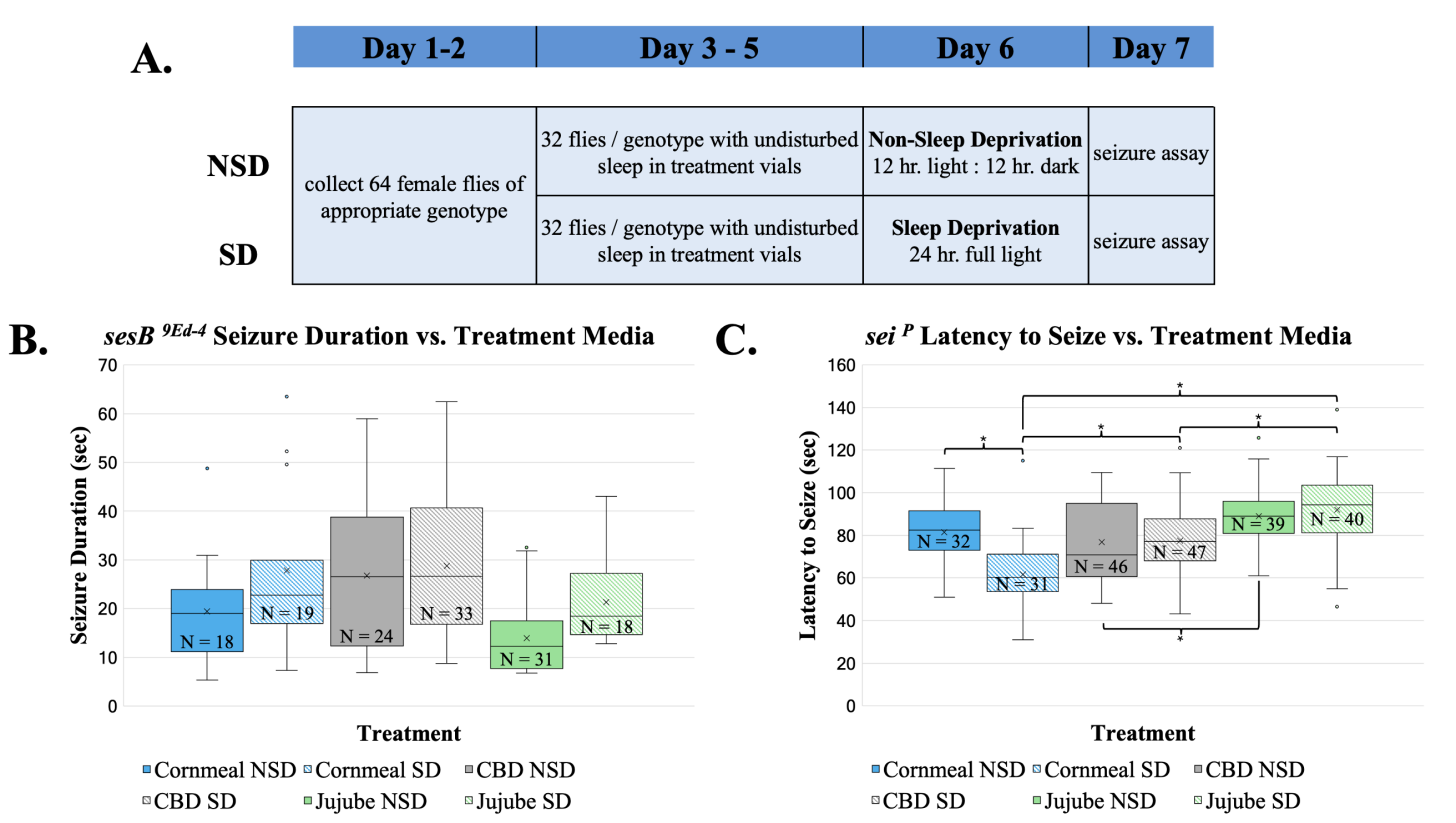
**A)** The schedule of sleep deprivation and seizure assays for all fly genotypes. Female flies were collected in days 1-2 and placed in individual vials on either treatment or control food for days 3-5. On day 6, half of the collected flies were subjected to sleep deprivation (SD), and the other half were allowed to maintain their natural sleep pattern (NSD). Then, on day 7, the appropriate seizure assay was performed. **B)** Seizure duration for *sesB ^9Ed-4^* heterozygous flies fed standard cornmeal media, CBD, and *Z. jujube* media. Sleep deprivation increased seizure duration across all treatments (F _(1, 137)_ =10.99, p=0.0012) and *Z. jujube* treatment reduced seizure duration as compared to CBD treatment (t=4.43, p=0.0001) and control media (t=2.93, p=0.012) regardless of sleep state. **C)** Latency to seize for *sei ^P^* mutants fed standard cornmeal media, CBD, and *Z. jujube* media. CBD and *Z. jujube* both increased the latency to seize in SD *sei ^P^* flies compared to SD flies fed standard cornmeal media (CBD: t=4.20, p=0.006; *Z. jujube* : t=-7.96, p<0.0001). &nbsp;Flies treated with *Z. jujube* had increased latency to seizure as compared to CBD-treated flies for both sleep conditions (NSD: t=-3.59, p=0.006; SD: t=-4.34, p=0.0003). Asterisks indicate statistically significant comparisons.

## Description

Epilepsy and seizures are serious health concerns, and more effective treatments are needed to support the affected population (Shneker and Fountain 2003). Currently approved medications for epilepsy vary in effectiveness; for instance, approximately one-third of epilepsy patients have drug-resistant epilepsy, meaning their seizures do not respond to medication (Löscher et al., 2020).

Epilepsy has been shown to reduce both the duration and quality of sleep, which in turn increases the likelihood of seizures, leading to longer, more severe episodes and a self-reinforcing cycle (Piperidou et al., 2008; Roliz and Kothare 2022). This demonstrates the bidirectional relationship between sleep deprivation and seizures (Bonilla-Jamie et al., 2021; Stirling et al., 2023). Using two separate seizure mutant genotypes in *Drosophila melanogaster* , Lucey et al. (2015) demonstrated that this relationship holds true in flies by showing that sleep deprivation increased seizure duration and decreased latency to seizure – defined as the time to paralysis onset. That work provides a model system to test potential drugs to treat epilepsy and reduce seizure susceptibility and severity.

This study used two *D. melanogaster* mutants that are commonly used to study seizures. The first, *stress-sensitive B* ( *sesB ^9Ed-4^* ), exhibits seizure-like behavior and paralysis following mechanical stress, such as vortexing. This is due to a mutation in the *sesB* gene that encodes an adenine nucleotide translocase (ANT), which has a high degree of sequence similarity to mammalian ANTs (Louvi and Tsitilou 1992; Zhang et al., 1999). Though mutations in human ANT are not directly linked with human seizure disorders, experimental evidence has demonstrated that *sesB* expression is altered in a *Drosophila* model of Dub15q epilepsy, a human genetic disorder associated with drug-resistant epilepsy (Hope et al., 2020). The second mutant, *sei ^P^* , displays similar seizure behavior in response to temperature stress, such as immersion in a warm water bath. This mutation affects a gene that encodes a voltage-gated potassium channel, and it is directly orthologous to the human ether-a-go-go related gene (hERG; Jackson et al., 1985; Titus et al., 1997). hERG is associated with the cardiac Long QT Syndrome (LQTS) in humans (Curran et al., 1995). Many LQTS patients also experience seizures, and work in *Drosophila* (Hill et al., 2019) as well as mammalian systems (Warmke et al., 1994; Chiesa et al., 1997; Wymore et al., 1997; Sacco et al., 2003; Johnson et al., 2009) indicate that both orthologs are expressed and function in the nervous system. The *sei ^P^* allele leads to a shorter latency to seizure and a lower temperature threshold for temperature stress-induced paralysis (Zheng et al., 2014).

Two prospective antiepileptic drugs (AEDs) include cannabidiol (CBD) and *Zizyphus jujube* extract. CBD has demonstrated anticonvulsant effects in various seizure models, including increasing the seizure threshold and reducing seizure duration in electroshock-induced seizures in mice (Chesher and Jackson 1974; Jones et al., 2012; Devinsky et al., 2024). Unlike many AEDs, CBD has minimal impact on motor function, protects against neuronal loss, acts as an antioxidant, and does not accelerate neural signaling. Instead, it reduces chemically induced seizures, making it a promising candidate for treating both partial and generalized seizures (Jones et al., 2012; Golub and Reddy 2021).

*Z. jujube* is commonly referred to as a Chinese date, and it is used in the practice of traditional Chinese medicine. *Z. jujube* has been shown to treat fatigue and sleep deprivation because of its flavonoid antioxidants that reduce stress (Agrawal et al., 2023). A study was conducted to investigate the anticonvulsant effects of *Z. jujube* extract on chemically and electrically induced seizures in rats. When rats were administered maximum electric shock that induced a seizure, a 1000 mg/kg dosage of *Z. jujube* produced significant protection from seizure (Pahuja et al., 2011).

Seizure activity was assessed in *sesB ^9Ed-4^* and *sei ^P^* mutants and the appropriate controls raised on standard cornmeal media and transferred to individual vials at the age of 1-2 days. When flies were transferred to individual vials, they were either maintained on standard media or treated with CBD or *Z. jujube* for three days ( Fig. 1A ) *.* Female flies were used to maintain consistency with previously published experiments (Lucey et al., 2015). Control flies, CS and *w ^1118^* did not exhibit seizures, so the data are not included in the figures or statistical analyses.

An Aligned Rank Transform (ART) test was used to compare the experimental groups because not all the data sets had a normal distribution. The ART main effects testing indicated that both treatment (F _(2, 137)_ =10.2, p=7.6x10 ^-5^ ) and sleep condition (F _(1, 137)_ =10.99, p=0.0012) had a significant effect on the duration of seizures in *sesB ^9Ed-4^* flies ( Fig. 1B ), while there was no significant interaction between the two factors (F _(2, 137)_ =0.41, p=0.67). As expected, sleep deprivation led to an increase in seizure duration across all treatments for the *sesB ^9Ed-4^* flies. The effect of sleep deprivation is consistent with previously published results (Lucey et al., 2015). Post-hoc ART-C results indicate that treatment with *Z. jujube* decreases seizure duration across both sleep conditions as compared to the control media (t=2.93, p=0.012) and CBD treatment (t=4.43, p=0.0001). CBD treatment did not have a significant effect on seizure duration as compared to controls (t=1.12, p=0.79). The *Z. jujube* results are consistent with our hypothesis, though is it surprising that CBD did not have a similar effect given previous results in other organisms (Golub and Reddy 2021). These results suggest that *Z. jujube* treatment counteracted the effects of sleep deprivation in the *sesB ^9Ed-4^* genotype, reducing seizure duration to a similar level to untreated flies that were not sleep-deprived.

A similar experiment was performed with *sei ^P^* flies raised on control, CBD or *Z. jujube* treatment media. The ART main effects testing with the *sei ^P^* experiment indicated that treatment had a significant effect on latency to seize (F _(2, 229)_ =29.37, p=4.43x10 ^-12^ ), and there was a significant interaction between treatment and sleep state (F _(2, 229)_ =12.81, p=5.29x10 ^-6^ ) even though sleep state alone did not have a significant effect across all treatments (F _(1, 229)_ =3.47, p=0.064). Given the interaction between treatment and sleep state, ART-C post-hoc analysis included pair-wise comparisons between all the experimental groups. As expected, sleep deprivation decreased latency to seize in flies fed control media ( Fig. 1C, blue bars; t=4.99, p<0.0001). SD flies fed either CBD or *Z. jujube* media had significantly longer latency to seizure than those fed standard cornmeal media ( Fig. 1C, striped bars; CBD: t=4.20, p=0.006; *Z. jujube* : t=-7.96, p<0.0001). These findings indicate that both treatments effectively mitigated the effects of sleep deprivation in the *sei ^P^* genotype, increasing seizure latency. Additionally, *sei ^P^* flies treated with *Z. jujube* showed increased latency to seize as compared with flies treated with CBD for both sleep states ( Fig. 1C, grey and green bars; NSD: t=-3.59,p=0.006; SD: t=-4.34, p=0.0003), suggesting that *Z. jujube* may have greater anti-convulsant activity than CBD.

Results from this study support the hypothesis that natural products such as CBD or *Z. jujube* possess potential anticonvulsant properties that reduce seizure duration and slow the onset of seizure. This work demonstrates that these drugs are effective in *D. melanogaster,* which provides a model system in which future experiments can explore their mechanism of action. Interestingly, CBD treatment was ineffective in the *sesB ^9Ed-4^* mutants, but it was effective in the sleep-deprived *sei ^P^* mutants. Differences in efficacy may allow researchers to better understand the mechanisms of these treatments.

## Methods

**Fly Stocks/Strains.** Canton-S, *sesB ^9Ed-4^* , *w ^1118^* , and *sei ^P^* flies were obtained through the Bloomington Drosophila Stock Center. Female flies from the CS, *w ^1118^* , and *sei ^P^* stocks were collected and put on control or treatment media. For the *sesB ^9Ed-4^* experiments, a cross between *sesB ^9Ed-4^* virgin females and CS males was performed to obtain heterozygous *sesB ^9Ed-4^* females without the balancer chromosome.&nbsp; Female offspring with wild-type eyes were collected and put into individual vials with the appropriate media. CS flies were used as the wild-type control for *sesB9 ^Ed-4^* , and *w ^1118^* flies were used as the control for *sei ^P^* (Zheng et al., 2014; Lucey et al., 2015).

**Control & Treatment Food.** The control food was Nutri-Fly Bloomington Formulation (Genesee Scientific) prepared according to the manufacturer’s instructions. The treatment media contained 100 mM CBD (Abdulla 2021) or 1% *Z. jujube* extract made as described by Hou et al. (2023).

**Sleep Deprivation.** Sleep deprivation was achieved by using the Sleep Nullifying Apparatus (SNAP) and 24 hours of ambient light (Melnattur et al., 2020). and was initiated at ZT 12. The SNAP rotated from +60º to -60º roughly every two seconds. Non-sleep-deprived flies were maintained with a 12-hour light:12-hour dark cycle without physical perturbation (Day 6, see Fig. 1A ). This protocol was demonstrated to increase seizure susceptibility in flies by Lucey, et al. (2015).

**Seizure Assays.** Seizure assays were performed as described by Lucey, et al. (2015). After sleep deprivation, the CS and *sesB ^9Ed-4^* heterozygous flies were transferred to individual vials without food and vortexed on the maximum setting for 10 seconds. After vortexing, the time it took for each fly to resume normal activity, such as standing upright or climbing the sides of the vial, was recorded as the seizure duration. Similarly, after sleep deprivation, the *w ^1118^* and *sei ^P^* mutants were put into individual vials without food and submerged in a 39ºC water bath. The time at which the fly was paralyzed was recorded as the latency to seizure.

**Statistical Analysis.** The Shapiro Normality Test was applied to all data sets for both genotypes, and some sets were not consistent with normality. To detect differences in seizure duration and seizure latency, an Aligned Rank Transform (ART) test was performed using the ARTool package in R (Wobbrock et al., 2011). ART is a non-parametric equivalent to a two-factor ANOVA. Post-hoc analyses were performed using the ART-C tool (Elkin et al., 2021) using the Bonferroni method to correct for multiple comparisons and the p-values reported in the text reflect the adjusted values. For the *sesB ^9Ed-4^* post-hoc analysis, treatments were compared across both sleep conditions because the main effects analysis indicated no interaction between the sleep condition and treatment type. In contrast, the main effects analysis for the *sei ^P^* data indicated an interaction between sleep condition and treatment, so pairwise comparisons were performed to determine the interactions.

## Reagents

**Table:** 

*Drosophila* strains	Genotype	Identifier	Available from
* sei ^P^*	w ^1118^ ; P{w ^+mC^ =EPg}sei ^HP21840^	BDSC_21935	Bloomington Drosophila Stock Center
* w ^1118^*	w ^1118^	BDSC_3605	Bloomington Drosophila Stock Center
* sesB ^9Ed-4^*	y ^1^ sesB ^9Ed-4^ v ^1^ f ^1^ /FM6	BDSC_8863	Bloomington Drosophila Stock Center
CS	Canton-S	BDSC_64349	Bloomington Drosophila Stock Center
